# Longitudinal Changes in Body Composition and Fluid Distribution During Chemotherapy in Breast Cancer Patients: A Prospective Single-Center Longitudinal Observational Study Using Bioimpedance Spectroscopy

**DOI:** 10.3390/jcm15124556

**Published:** 2026-06-12

**Authors:** Aysun Fatma Akkuş, Gökhan Öztürk, Ömer Ferudun Akkuş, İlhan Kurultak, Tayyip İlker Aydın, Ahmet Küçükarda, Muhammet Bekir Hacıoğlu, Sernaz Topaloğlu, Bülent Erdoğan

**Affiliations:** 1 Department of Medical Oncology, Faculty of Medicine, Trakya University, 22030 Edirne, Türkiye; gokymd@gmail.com (G.Ö.); ilker6125@gmail.com (T.İ.A.); ahmetkucukarda22@gmail.com (A.K.); mbekirhacioglu@yahoo.com (M.B.H.); sernaz.uzunoglu@gmail.com (S.T.); berdoga@hotmail.com (B.E.); 2 Department of Cardiology, Edirne Sultan 1. Murat State Hospital, 22030 Edirne, Türkiye; akkusferudun@gmail.com; 3 Department of Nephrology, Faculty of Medicine, Trakya University, 22030 Edirne, Türkiye; ilhankurultak@yahoo.co.uk

**Keywords:** breast cancer, chemotherapy, body composition, bioelectrical impedance spectroscopy, fluid distribution, lean tissue mass, extracellular water, sarcopenia

## Abstract

**Background**: Anthracycline- and taxane-based chemotherapy regimens are widely used in the treatment of breast cancer; however, their effects on body composition and fluid distribution are not fully elucidated. Conventional assessment methods are often insufficient to distinguish true tissue changes from treatment-related fluid shifts. The primary objective of this study was to evaluate longitudinal changes in body composition and fluid distribution during chemotherapy in breast cancer patients using bioelectrical impedance spectroscopy. The secondary objective was to investigate the impact of anthracycline and docetaxel exposure on these changes and to identify patterns suggestive of masked sarcopenia. **Methods**: This prospective, single-center, longitudinal observational study was conducted between October 2024 and October 2025. Follow-up assessments at 3 and 6 months were completed by October 2025. A total of 51 female breast cancer patients undergoing systemic chemotherapy were evaluated using multifrequency bioelectrical impedance spectroscopy (BCM^®^). Measurements were performed at baseline, 3 months, and 6 months. Changes in total body water (TBW), extracellular water (ECW), intracellular water (ICW), extracellular-to-intracellular water ratio (E/I), lean tissue mass (LTM), adipose tissue mass (ATM), and volume status were analyzed longitudinally and according to treatment exposure. **Results**: The cohort consisted of 51 women (median age, 55 years), of whom 70.6% were postmenopausal, and the majority had stage II–III disease. While TBW remained stable, significant alterations in fluid distribution and body composition were observed. ECW increased, and ICW decreased, resulting in a significant rise in the E/I ratio. LTM declined significantly, particularly during the first 3 months, whereas ATM showed a gradual increase. Volume status increased progressively over time, indicating fluid accumulation. Anthracycline exposure was associated with greater reductions in LTM, while docetaxel treatment was linked to significant increases in extracellular fluid and volume, especially during the 3–6-month interval. At 6 months, a median increase of +1100 mL in volume was observed alongside a decrease in muscle mass (−1.4 kg), consistent with a pattern of masked sarcopenia. **Conclusions**: Chemotherapy in breast cancer patients is associated with concurrent muscle loss and fluid redistribution, which may obscure clinically relevant changes in body composition. Bioelectrical impedance spectroscopy enables differentiation between fluid and tissue compartments and provides a more accurate assessment than conventional methods. Early recognition of these changes may facilitate timely nutritional support and appropriate fluid management strategies.

## 1. Introduction

Breast cancer is the most frequently diagnosed malignancy among women worldwide, accounting for 11.7% of all cancer cases according to 2020 data [1]. Disease management is a multidisciplinary process that varies according to clinical stage and biological subtype, encompassing neoadjuvant, adjuvant, and metastatic/palliative systemic therapies. In this context, anthracycline- and taxane-based chemotherapy regimens constitute the cornerstone of treatment for both curative and palliative purposes [2,3]. Despite the significant improvement in survival rates, long-term treatment-related adverse effects, particularly those affecting body composition and metabolic balance, are gaining increasing clinical importance.

Body mass index (BMI), which is widely used in clinical practice, is insufficient for assessing tissue-level changes that occur during the course of chemotherapy. In patients undergoing chemotherapy, sarcopenic obesity, characterized by a decrease in lean tissue mass (LTM) and an increase in adipose tissue mass (ATM), is frequently observed, even when overall body weight remains stable [4,5,6,7]. This condition, defined as “masked sarcopenia,” is associated with an increased risk of grade 3/4 chemotherapy-related toxicity, reduced functional capacity, decreased quality of life, and poorer overall survival [8,9,10]. Furthermore, since chemotherapy dosing is predominantly calculated based on Body Surface Area (BSA), which does not reflect tissue distribution, drug toxicity may be more pronounced in patients with low muscle mass [2].

In addition to alterations in body composition, taxane- and anthracycline-based chemotherapy regimens, particularly docetaxel, have been associated with systemic fluid retention prior to the onset of clinically overt peripheral edema [11]. The resultant increase in extracellular water (ECW) may contribute to elevated cardiac afterload, thereby promoting rises in B-type natriuretic peptide (BNP) levels, a recognized biomarker of myocardial stress [12]. Accordingly, the early identification of subclinical fluid overload is of considerable clinical relevance, as it enables the timely implementation of preventive interventions, such as diuretic therapy or sodium restriction, and may mitigate the risk of chemotherapy-related cardiotoxicity [13,14].

Bioelectrical impedance spectroscopy (BIS), as implemented in the Body Composition Monitor (BCM^®^, Fresenius Medical Care), enables the assessment of body composition through multifrequency measurements, categorizing it into three principal compartments: fat mass, lean tissue mass, and overhydration (OH) [15,16]. This technology allows for the objective determination of the patient’s true “dry weight” by minimizing the masking effect of edema on muscle loss and facilitates the early detection of reductions in muscle mass [13,16]. Consequently, individualized nutritional support can be initiated prior to the development of overt muscle wasting, and fluid balance can be managed proactively.

It has been reported in the literature that the prevalence of malnutrition among cancer patients ranges from 20% to 87%, and that this condition directly impacts survival [17,18]. However, the inclusion of patients who were not at risk of malnutrition at baseline, according to the Global Leadership Initiative on Malnutrition criteria, indicates that the observed changes in body composition and fluid status during follow-up are more likely attributable to chemotherapy regimens rather than to pre-existing nutritional impairment.

The aim of this study is to longitudinally evaluate the effects of chemotherapy regimens with or without anthracyclines and taxanes on body composition and fluid dynamics in breast cancer patients, using BCM measurements at baseline, 3 months, and 6 months, and to elucidate treatment-related physiological changes associated with different regimens.

## 2. Materials and Methods

### 2.1. Study Design and Patient Population

This prospective longitudinal observational study included 51 female patients diagnosed with breast cancer and followed at the Department of Medical Oncology, Trakya University Faculty of Medicine. Patient recruitment and baseline assessments were conducted between October 2024 and April 2025. Follow-up assessments at 3 and 6 months were completed by October 2025. Patients who underwent serial bioimpedance analysis measurements at baseline, 3 months, and 6 months during systemic chemotherapy were eligible for inclusion. All consecutively enrolled patients who met the eligibility criteria completed the scheduled assessments and were included in the final analysis. No patients were excluded because of missing data, and no loss to follow-up occurred during the study period.

Exclusion criteria were defined as the presence of conditions that could significantly affect body composition or fluid distribution, including severe cardiac disease, active infection, chronic pulmonary disease, the presence of metallic implants or prostheses interfering with bioimpedance measurements, limb amputation, and incomplete clinical or bioimpedance data.

All patients received standard chemotherapy regimens, and treatment exposure was categorized according to anthracycline and docetaxel use. Clinical, demographic, and treatment-related data were obtained from electronic medical records. Patients with HER2-positive disease received trastuzumab-based anti-HER2 therapy according to current treatment guidelines and institutional treatment protocols. Trastuzumab treatment was administered concurrently with or sequentially after chemotherapy, depending on the treatment setting and clinical indication. Menopausal status was defined according to standard clinical criteria. Women with ≥12 consecutive months of amenorrhea without any pathological or physiological cause were classified as postmenopausal. Women with ongoing menstrual cycles or amenorrhea lasting less than 12 months were classified as premenopausal.

Nutritional status at baseline was assessed according to the Global Leadership Initiative on Malnutrition (GLIM) criteria. The GLIM framework includes a two-step approach consisting of initial risk screening followed by diagnostic assessment based on phenotypic (non-volitional weight loss, low body mass index, reduced muscle mass) and etiologic (reduced food intake or assimilation, disease burden/inflammation) criteria. In this study, none of the patients met the diagnostic criteria for malnutrition at baseline, indicating that the study population consisted of individuals without pre-existing malnutrition, thereby allowing evaluation of chemotherapy-related changes independent of baseline nutritional impairment.

The study was conducted in accordance with the Declaration of Helsinki and approved by the Ethics Committee of Trakya University Faculty of Medicine (approval no. 2024/373, dated 23 September 2024). Written informed consent was obtained from all patients prior to inclusion.

#### Bioimpedance Analysis

Body composition and fluid parameters were assessed using the BCM^®^ Body Composition Monitor (Fresenius Medical Care AG & Co., Bad Homburg, Germany), a multifrequency bioimpedance spectroscopy device. Measurements were performed at three predefined time points: prior to initiation of chemotherapy (baseline), at 3 months, and at 6 months during treatment.

The parameters obtained included total body water (TBW), extracellular water (ECW), intracellular water (ICW), and volume status (VS). In addition, lean tissue mass (LTM) and adipose tissue mass (ATM) were derived from the device outputs and recorded for analysis.

All measurements were conducted under standardized conditions to minimize variability. Patients were instructed to fast for at least 6 h prior to measurement, avoid caffeine, alcohol, and strenuous physical activity, and refrain from smoking on the day of assessment.

Measurements were performed with patients in the supine position after a short rest period, in accordance with the manufacturer’s recommendations. Patients were asked to remove all metallic accessories before the procedure. Age, height, and body weight were recorded at each time point and entered into the device prior to measurement.

To ensure consistency, all measurements were performed using the same device and standardized protocol at each time point.

## 3. Definition of Body Composition Parameters and Outcome Measures

Changes in body composition parameters were evaluated across three predefined time intervals: baseline to 3 months, 3 to 6 months, and baseline to 6 months.

Extracellular fluid volume, lean tissue mass (LTM), and adipose tissue mass (ATM) were analyzed both as absolute values and as changes over time. Delta values were calculated for each parameter as follows: Δ_1_ (0–3 months), Δ_2_ (3–6 months), and Δ_3_ (0–6 months).

For categorical analyses, changes in parameters were classified according to their direction. An increase in extracellular fluid volume was defined as ΔECF > 0, reflecting fluid accumulation, whereas no increase was defined as ΔECF ≤ 0.

For lean tissue mass and adipose tissue mass, a decrease was defined as Δ < 0, and no decrease was defined as Δ ≥ 0.

Treatment exposure variables were defined based on chemotherapy regimens. Patients were categorized according to anthracycline exposure (yes/no) and docetaxel exposure (yes/no) for subgroup analyses.

### Statistical Analysis

Statistical analyses were performed using IBM SPSS Statistics for Windows, version 22 (IBM Corp., Armonk, NY, USA).

Continuous variables were assessed for normality using the Shapiro–Wilk test. As most variables were not normally distributed, data were expressed as median values and analyzed using nonparametric methods. Categorical variables are presented as frequencies and percentages.

Comparisons of repeated measurements (baseline, 3 months, and 6 months) were performed using the Friedman test. When a significant overall difference was detected, post-hoc pairwise comparisons were conducted using the Wilcoxon signed-rank test.

For comparisons between two independent groups (anthracycline exposure and docetaxel exposure), the Mann–Whitney U test was used for continuous variables, and the chi-square test or Fisher’s exact test was applied for categorical variables, as appropriate.

Changes in body composition parameters were further analyzed using delta values (Δ_1_: 0–3 months; Δ_2_: 3–6 months; Δ_3_: 0–6 months). Group comparisons were primarily performed using these delta variables to evaluate treatment-related effects.

Associations between continuous variables were assessed using Spearman’s rank correlation analysis.

All statistical tests were two-sided, and a *p*-value < 0.05 was considered statistically significant.

## 4. Results

### 4.1. Patient Characteristics

A total of 51 female patients were included in the study. Most patients had never smoked (68.6%) and had an ECOG performance status of 0 (94.1%).

In terms of pathological diagnosis, the predominant histological subtype was invasive carcinoma (n = 43, 84.3%), followed by lobular carcinoma (n = 6, 11.8%) and mixed carcinoma (n = 2, 3.9%). Molecular subtype distribution revealed that 25 patients (49.0%) were HR-positive/HER2-negative, 14 (27.5%) were HR-positive/HER2-positive, 2 (3.9%) were HR-negative/HER2-positive, and 10 (19.6%) were triple-negative.

Regarding menopausal status, 36 patients (70.6%) were postmenopausal, and 15 (29.4%) were premenopausal. At diagnosis, most patients were in stage II or III disease (each n = 21, 41.2%), while 5 patients (9.8%) had stage I and 4 patients (7.8%) had stage IV disease. Anthracycline-based chemotherapy was administered in 29 patients (56.9%), whereas 22 patients (43.1%) did not receive anthracycline treatment (Table 1).

### 4.2. Overall Changes in Body Composition and Fluid Distribution

During systemic chemotherapy, a complex pattern of body composition changes was observed (Table 2, Table 3, Table 4 and Table 5). While total body water (TBW) remained relatively stable over time, significant alterations in fluid distribution and tissue composition were detected. In particular, extracellular water (ECW) increased, whereas intracellular water (ICW) decreased, resulting in a significant rise in the extracellular-to-intracellular water (E/I) ratio.

In parallel, lean tissue mass (LTM) showed a significant decline, predominantly during the early phase of treatment, whereas adipose tissue mass (ATM) demonstrated a gradual increase over time. Bioimpedance-derived volume status also increased significantly, indicating progressive fluid accumulation.

Delta analyses further demonstrated that most of these changes occurred within the first 3 months of treatment, followed by a relative stabilization phase (Table 3). Treatment-stratified analyses revealed that anthracycline exposure was associated with greater reductions in LTM, whereas docetaxel use was linked to increased volume expansion and fluid redistribution (Table 4 and Table 5).

**Figure 1:**
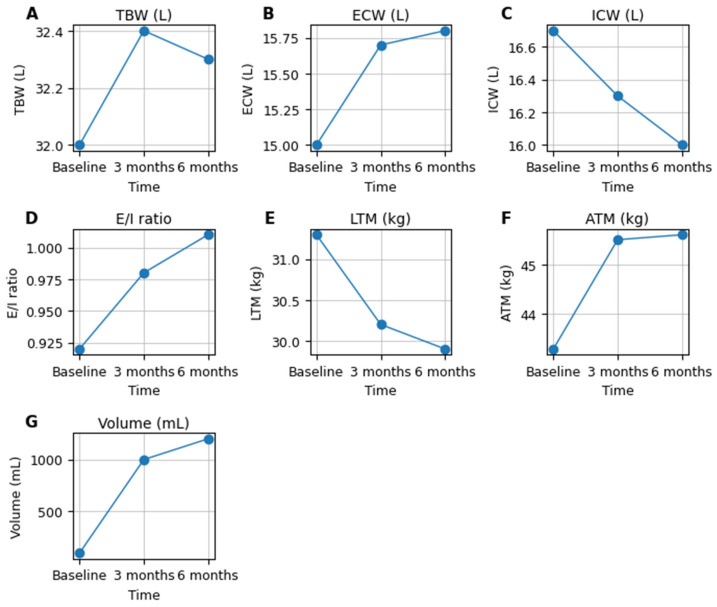
Graphical representation of longitudinal changes in body composition parameters: (**A**) total body water (TBW), (**B**) extracellular water (ECW), (**C**) intracellular water (ICW), (**D**) extracellular-to-intracellular water ratio (E/I), (**E**) lean tissue mass (LTM), (**F**) adipose tissue mass (ATM), and (**G**) volume status.

Taken together, these findings indicate a shift in body composition characterized by extracellular fluid expansion, intracellular fluid reduction, loss of lean mass, and gain in adipose tissue during chemotherapy. These longitudinal changes in body composition parameters are graphically illustrated in Figure 1.

### 4.3. Changes in Extracellular Fluid Volume

Changes in extracellular fluid volume were evaluated across three time intervals (0–3 months, 3–6 months, and 0–6 months). An increase in extracellular fluid volume was observed in the majority of patients at all time points, with increases detected in 84.3%, 70.6%, and 86.3% of patients for ΔECF_1_, ΔECF_2_, and ΔECF_3_, respectively.

A significant overall difference in extracellular fluid volume changes over time was observed (χ^2^ = 36.57, *p* < 0.001). Pairwise comparisons showed that extracellular fluid volume changes were significantly lower in the 3–6-month interval compared to the 0–3-month interval (Z = −3.341, *p* = 0.001), followed by a significant increase from 3–6 months to 0–6 months (Z = −5.161, *p* < 0.001). Extracellular fluid volume at 6 months remained significantly higher compared to baseline (Z = −3.197, *p* = 0.001).

A strong positive association was observed between ΔECF_1_ and ΔECF_3_ (ρ = 0.764, *p* < 0.001) and a moderate association between ΔECF_2_ and ΔECF_3_ (ρ = 0.578, *p* < 0.001), whereas no significant association was observed between ΔECF_1_ and ΔECF_2_ (*p* = 0.590).

When stratified according to docetaxel exposure, patients receiving docetaxel exhibited significantly greater increases in extracellular fluid volume during the 3–6-month interval (ΔECF_2_; *p* = 0.015) and over the entire 0–6-month period (ΔECF_3_; *p* = 0.033), whereas no significant difference was observed during the early period (ΔECF_1_; *p* = 0.131).

### 4.4. Changes in Lean Tissue Mass (LTM)

Lean tissue mass was evaluated at baseline, 3 months, and 6 months. A significant difference in LTM across time points was observed (χ^2^ = 17.92, *p* < 0.001).

Pairwise comparisons showed a significant decrease in LTM from baseline to 3 months (Z = −2.869, *p* = 0.004), while no significant change was observed between 3 and 6 months (Z = −0.786, *p* = 0.432). LTM at 6 months remained significantly lower compared to baseline (Z = −2.988, *p* = 0.003).

A decrease in LTM was observed in 74.5% of patients during the 0–3-month interval (ΔLTM_1_) and in 74.5% over the entire 0–6-month period (ΔLTM_3_). During the 3–6-month interval (ΔLTM_2_), 52.9% of patients demonstrated a decrease, while 47.1% showed an increase.

When stratified by anthracycline exposure, no significant differences were observed in absolute LTM values at baseline (*p* = 0.140), 3 months (*p* = 0.909), or 6 months (*p* = 0.690). However, greater reductions in LTM were observed in patients receiving anthracycline during the 0–3-month interval (ΔLTM_1_; *p* = 0.013) and over the entire 0–6-month period (ΔLTM_3_; *p* = 0.015), whereas no significant difference was observed during the 3–6-month interval (ΔLTM_2_; *p* = 0.447).

When stratified according to docetaxel exposure, no significant differences were observed in LTM changes between groups at any time interval (ΔLTM_1_, *p* = 0.117; ΔLTM_2_, *p* = 0.483; ΔLTM_3_, *p* = 0.094).

### 4.5. Changes in Adipose Tissue Mass (ATM)

Adipose tissue mass was evaluated at baseline, 3 months, and 6 months. A significant overall difference in ATM across time points was observed (χ^2^ = 8.355, *p* = 0.015).

Pairwise comparisons showed no statistically significant differences between baseline and 3 months (Z = −1.744, *p* = 0.081), between 3 and 6 months (Z = −0.058, *p* = 0.954), or between baseline and 6 months (Z = −1.659, *p* = 0.097).

ATM increased in 70.0% of patients during the 0–3-month interval (ΔATM_1_) and in 62.7% over the entire 0–6-month period (ΔATM_3_). During the 3–6-month interval (ΔATM_2_), 54.0% of patients demonstrated a decrease, while 46.0% showed an increase.

When stratified according to docetaxel exposure, no significant differences were observed in ATM changes between groups at any time interval (ΔATM_1_, *p* = 0.114; ΔATM_2_, *p* = 0.215; ΔATM_3_, *p* = 0.420).

When stratified by anthracycline exposure, no significant differences were observed in ATM changes between groups at any time interval (ΔATM_1_, *p* = 0.135; ΔATM_2_, *p* = 0.464; ΔATM_3_, *p* = 0.213).

## 5. Discussion

This study evaluates the longitudinal effects of anthracycline- and taxane-based chemotherapy regimens on body composition and fluid dynamics in breast cancer patients using multifrequency bioelectrical impedance spectroscopy (BCM^®^). Our findings indicate that although total body water (TBW) remained relatively stable throughout the treatment period, significant alterations occurred in fluid distribution and tissue composition. Specifically, an increase in extracellular water (ECW) accompanied by a decrease in intracellular water (ICW) resulted in a significant rise in the extracellular-to-intracellular water ratio (E/I). In parallel, a significant reduction in lean tissue mass (LTM) was observed, while adipose tissue mass (ATM) showed a tendency to increase over time. In addition, an increase in volume status was detected, suggesting progressive fluid accumulation. Notably, these changes were more pronounced during the first three months of treatment, followed by a relative stabilization phase.

In patients with breast cancer, the observed reduction in lean tissue mass (LTM) alongside a concomitant increase in adipose tissue mass (ATM) during chemotherapy reflects the process of “metabolic remodeling” described in the literature [4,5,6]. These alterations at the tissue level are indicative of the development of sarcopenic obesity, characterized by progressive muscle loss accompanied by a relative increase in adiposity [19,20,21].

Furthermore, the significant elevation in the extracellular-to-intracellular water ratio (E/I) observed in our cohort may reflect alterations in cellular integrity and fluid homeostasis, potentially driven by treatment-related inflammation and endothelial dysfunction [22,23].

Collectively, our findings are consistent with previous reports demonstrating increases in fat mass and reductions in lean mass during anthracycline- and taxane-based chemotherapy, further supporting the growing body of evidence that systemic anticancer therapies exert deleterious effects on body composition beyond those captured by conventional anthropometric measures [24,25].

The longitudinal analysis of our study demonstrated that anthracycline- and taxane-based chemotherapy regimens exert distinct and partially divergent effects on body composition and fluid balance. In particular, patients exposed to anthracyclines showed a more pronounced decline in lean tissue mass (LTM) both during the early phase of treatment (0–3 months, ΔLTM_1_) and over the entire follow-up period (0–6 months, ΔLTM_3_) (*p* = 0.013 and *p* = 0.015, respectively). Anthracycline-treated patients showed a median ΔLTM_3_ of −2.3 kg compared with −0.2 kg in the non-anthracycline group (*p* = 0.015).

Consistent with the existing literature, this finding may be attributed to anthracycline-induced oxidative stress, mitochondrial damage at the myocyte level, and systemic inflammation [13,26,27]. Mazzuca et al. (2018) demonstrated that loss of lean tissue mass in early-stage breast cancer patients receiving anthracyclines is an independent predictor of severe toxicity and is characterized by progressive muscle degradation driven by suppressed protein synthesis and enhanced proteolysis [28]. Similarly, evidence from the literature indicates longitudinal declines in skeletal muscle mass and body cell mass associated with anthracycline-based (FAC) regimens [29], supporting our findings.

In our study, patients treated with docetaxel demonstrated a significant increase in extracellular fluid and total body volume, particularly during the later phases of treatment (months 3–6) (*p* = 0.015). By month six, the median increase in volume was 1500 mL in the docetaxel group compared with 350 mL in the non-docetaxel group (*p* = 0.033). This observation is consistent with the known effects of taxanes on endothelial integrity and capillary permeability [30,31]. The so-called “capillary leak” phenomenon likely underlies this process, leading to a shift of fluid from the intravascular to the interstitial space [32,33].

When interpreted in the context of DXA-based studies, which remain the most commonly used method for body composition assessment in the literature despite their limited ability to distinguish fluid shifts from true tissue changes, our findings provide a clearer characterization of fluid-related alterations [34,35]. Previous reports have suggested that increases in lean body mass during chemotherapy may reflect fluid retention rather than true tissue gain [36,37]. In line with this, the use of BCM^®^ in our study enabled differentiation between fluid and tissue compartments, demonstrating a decrease in muscle mass (−1.4 kg) alongside an increase in fluid volume (+1100 mL) at 6 months, consistent with a pattern of “masked sarcopenia.”

The role of corticosteroid premedication appears to be complex. Although it is routinely used to reduce hypersensitivity reactions and edema, its mineralocorticoid effects may promote sodium and water retention, potentially contributing to fluid accumulation [38,39]. In line with this, the increase in the extracellular-to-intracellular fluid ratio (from 0.92 to 1.01) in our study may reflect the combined impact of treatment-related toxicity and steroid exposure on cellular homeostasis.

Taken together, the fluid-retentive effect of docetaxel, which becomes more evident in the later stages of treatment, may obscure concurrent catabolic changes associated with anthracyclines. As a result, alterations in body composition may be underestimated when relying solely on conventional assessments [40,41,42]. These findings highlight the importance of monitoring fluid status using bioelectrical impedance spectroscopy (BIS), particularly in patients receiving docetaxel, to enable a more accurate evaluation of muscle loss and cardiovascular burden.

This study has several limitations. The relatively small sample size, single-center setting, observational design, and lack of a control group may limit the generalizability of the findings and preclude causal inferences. In addition, the marked imbalance in sample size between the docetaxel-treated (n = 44) and non-docetaxel (n = 6) subgroups may have limited the statistical power and robustness of subgroup analyses. Therefore, findings related to docetaxel-specific effects should be interpreted with caution. The non-uniform distribution of patients across disease stages, particularly the limited number of stage I and stage IV cases, may have influenced the interpretation of the results. Furthermore, although BCM^®^ provides a detailed assessment of fluid compartments, it remains an indirect measurement method and may be subject to measurement variability.

## 6. Conclusions

In conclusion, anthracycline- and taxane-based chemotherapy regimens are associated with significant alterations in body composition, characterized by a reduction in muscle mass accompanied by an increase in extracellular fluid. Importantly, docetaxel-related fluid retention may mask concurrent muscle loss, thereby complicating clinical assessment and potentially leading to underrecognition of sarcopenia. These findings suggest a possible association between docetaxel exposure and extracellular fluid expansion; however, confirmation in larger and more balanced cohorts is warranted.

These findings highlight the need to move beyond conventional assessment methods and adopt approaches capable of distinguishing fluid accumulation from true tissue changes. Early nutritional intervention may help attenuate muscle loss, whereas recognition of fluid overload should prompt appropriate clinical management, including fluid regulation, sodium restriction, and, when clinically indicated, diuretic therapy.

## Figures and Tables

**Table 1:** Baseline patient characteristics.

Baseline Characteristics	n (%)
Total patients	51
ECOG PS = 0	48 (94.1)
Never-smokers	35 (68.6)
Age, years, median (range)	55 (29–77)
BMI, kg/m^2^, median (IQR)	30.0 (25.0–33.8)
**Menopausal Status**	
Postmenopausal	36 (70.6)
Premenopausal	15 (29.4)
**Disease Stage**	
Stage I	5 (9.8)
Stage II	21 (41.2)
Stage III	21 (41.2)
Stage IV	4 (7.8)
**Histological Type**	
Invasive carcinoma	43 (84.3)
Lobular carcinoma	6 (11.8)
Mixed carcinoma	2 (3.9)
**Molecular Subtype**	
HR+/HER2−	25 (49.0)
HR+/HER2+	14 (27.5)
HR−/HER2+	2 (3.9)
Triple-negative	10 (19.6)
**Treatment Exposure**	
Anthracycline use	29 (56.9)
No anthracycline	22 (43.1)

ECOG, Eastern Cooperative Oncology Group; HR, hormone receptor; HER2, human epidermal growth factor receptor 2.

**Table 2:** Longitudinal changes in body composition parameters.

Parameter	Baseline	3 Months	6 Months	Δ (0–6 Months)	*p*-Value
TBW (L)	32.0	32.4	32.3	+0.3	0.616
ECW (L)	15.0	15.7	15.8	+0.8	<0.001
ICW (L)	16.7	16.3	16.0	−0.7	0.015
E/I ratio	0.92	0.98	1.01	+0.09	0.011
LTM (kg)	31.3	30.2	29.9	−1.4	<0.001
ATM (kg)	43.3	45.5	45.6	+2.3	0.015
Volume (mL)	100	1000	1200	+1100	<0.001

TBW, total body water; ECW, extracellular water; ICW, intracellular water; E/I ratio, extracellular-to-intracellular water ratio; LTM, lean tissue mass; ATM, adipose tissue mass; Δ, absolute change over time.

**Table 3:** Changes in body composition parameters.

Parameter	Δ_1_ (0–3 Months)	Δ_2_ (3–6 Months)	Δ_3_ (0–6 Months)
ATM (kg)	1.3 (4.9)	0.0 (1.7)	1.2 (8.7)
LTM (kg)	−1.2 (5.2)	0.0 (2.5)	−1.1 (5.6)
Volume (mL)	1000 (1200)	400 (700)	1400 (1700)
E/I ratio	0.058 (0.12)	0.013 (0.13)	0.081 (0.14)

Data are presented as medians (interquartile ranges). Δ_1_ represents changes from baseline to 3 months, Δ_2_ from 3 to 6 months, and Δ_3_ from baseline to 6 months. Abbreviations: ATM, adipose tissue mass; LTM, lean tissue mass; E/I ratio, extracellular-to-intracellular water ratio. Δ, change over time.

**Table 4:** Impact of anthracycline use on body composition changes.

Parameter	Non-Anthracycline (n = 22) Median (IQR)	Mean Rank	Anthracycline (n = 29) Median (IQR)	Mean Rank	*p*-Value *
ΔATM_1 (kg)	0.85 (5.77)	22.43	1.70 (6.85)	28.71	0.135
ΔATM_2 (kg)	0.00 (2.05)	24.25	0.10 (2.05)	27.33	0.464
ΔATM_3 (kg)	0.75 (5.97)	23.02	2.10 (11.10)	28.26	0.213
ΔVolume_1 (mL)	1000 (1825)	26.84	900 (1000)	25.36	0.725
ΔVolume_2 (mL)	500 (725)	26.48	400 (650)	25.64	0.841
ΔVolume_3 (mL)	1200 (1925)	26.75	1400 (1700)	25.43	0.753
ΔE/I_1	0.061 (0.13)	25.68	0.054 (0.12)	26.24	0.894
ΔE/I_2	0.010 (0.19)	26.48	0.014 (0.11)	25.64	0.842
ΔE/I_3	0.087 (0.20)	26.64	0.081 (0.12)	25.52	0.790
ΔLTM_1 (kg)	−0.40 (7.40)	31.93	−1.90 (5.00)	21.50	**0.013**
ΔLTM_2 (kg)	0.20 (2.87)	27.82	−0.70 (2.60)	24.62	0.447
ΔLTM_3 (kg)	−0.20 (4.00)	31.80	−2.30 (6.80)	21.60	**0.015**

* Data are presented as median values with interquartile ranges (IQRs) and mean ranks. Between-group comparisons were performed using the Mann–Whitney U test. ATM, adipose tissue mass; LTM, lean tissue mass; E/I ratio, extracellular-to-intracellular water ratio; Δ, absolute change over time; mL, milliliters.

**Table 5:** Impact of docetaxel treatment on body composition changes.

Parameter	Non-Docetaxel (n = 6) Median (IQR)	Mean Rank	Docetaxel (n = 44) Median (IQR)	Mean Rank	*p*-Value *
ΔATM_1 (kg)	−0.50 (9.75)	16.67	1.55 (5.28)	26.70	0.114
ΔATM_2 (kg)	0.15 (2.18)	32.42	−0.05 (2.03)	24.56	0.215
ΔATM_3 (kg)	1.10 (11.00)	21.00	1.30 (9.17)	26.11	0.420
ΔVolume_1 (mL)	350 (1100)	17.08	1000 (1300)	26.65	0.131
ΔVolume_2 (mL)	0 (475)	11.92	500 (600)	27.35	**0.015**
ΔVolume_3 (mL)	350 (1025)	13.58	1500 (1700)	27.13	**0.033**
ΔE/I_1	0.022 (0.11)	15.33	0.071 (0.15)	26.89	0.069
ΔE/I_2	−0.018 (0.15)	20.92	0.019 (0.12)	26.13	0.412
ΔE/I_3	−0.023 (0.15)	14.50	0.094 (0.12)	27.00	**0.049**
ΔLTM_1 (kg)	−0.25 (6.00)	34.25	−1.60 (5.13)	24.31	0.117
ΔLTM_2 (kg)	0.10 (0.90)	29.42	−0.40 (3.13)	24.97	0.483
ΔLTM_3 (kg)	−0.10 (6.57)	34.83	−1.85 (5.78)	24.23	0.094

* Data are presented as median values with interquartile ranges (IQRs) and mean ranks. Between-group comparisons were performed using the Mann–Whitney U test. Docetaxel refers to patients receiving docetaxel-containing chemotherapy regimens. ATM, adipose tissue mass; LTM, lean tissue mass; E/I ratio, extracellular-to-intracellular water ratio; Δ, absolute change over time; mL, milliliters.

## Data Availability

The data supporting the findings of this study are available within the article. Further inquiries may be directed to the corresponding author. Correspondence and requests for materials should be addressed to Aysun Fatma Akkuş (e-mail: aysunfatmadogan@gmail.com).
